# Ag as Cocatalyst and Electron-Hole Medium in CeO_2_ QDs/Ag/Ag_2_Se Z-scheme Heterojunction Enhanced the Photo-Electrocatalytic Properties of the Photoelectrode

**DOI:** 10.3390/nano10020253

**Published:** 2020-01-31

**Authors:** Lingwei Li, Hange Feng, Xiaofan Wei, Kun Jiang, Shaolin Xue, Paul K. Chu

**Affiliations:** 1 College of Science, Donghua University, Shanghai 201620, China; li.lingwei.pink@163.com (L.L.); 17721486315@163.com (H.F.);; 2 Department of Physics, Department of Materials Science and Engineering, and Department of Biomedical Engineering, City University of Hong Kong, Tat Chee Avenue, Kowloon, Hong Kong 999077, China

**Keywords:** CeO_2_ quantum dots, Ag_2_Se nanoflowers, Z-scheme, cocatalyst, photo-electrocatalysis

## Abstract

A recyclable photoelectrode with high degradation capability for organic pollutants is crucial for environmental protection and, in this work, a novel CeO_2_ quantum dot (QDs)/Ag_2_Se Z-scheme photoelectrode boasting increased visible light absorption and fast separation and transfer of photo-induced carriers is prepared and demonstrated. A higher voltage increases the photocurrent and 95.8% of tetracycline (TC) is degraded by 10% CeO_2_ QDs/Ag_2_Se in 75 minutes. The degradation rate is superior to that achieved by photocatalysis (92.3% of TC in 90 min) or electrocatalysis (27.7% of TC in 90 min). Oxygen vacancies on the CeO_2_ QDs advance the separation and transfer of photogenerated carriers at the interfacial region. Free radical capture tests demonstrate that •O_2_^−^, •OH, and h^+^ are the principal active substances and, by also considering the bandgaps of CeO_2_ QDs and Ag_2_Se, the photocatalytic mechanism of CeO_2_ QDs/Ag_2_Se abides by the Z-scheme rather than the traditional heterojunction scheme. A small amount of metallic Ag formed in the photocatalysis process can form a high-speed charge transfer nano channel, which can greatly inhibit the photogenerated carrier recombination, improve the photocatalytic performance, and help form a steady Z-scheme photocatalysis system. This study would lay a foundation for the design of a Z-scheme solar photocatalytic system.

## 1. Introduction

Pollution by drug antibiotics constitutes one of the major environmental problems and tetracycline (TC), which is a common drug with high antimicrobial activity against pathogenic bacteria, ranks second globally in terms of production and use [1,2]. Whereas, the poor absorption and low metabolic rate of TC by humans and animals lead to a discharge of a large amount of TC in the original form or metabolites into the environment [3,4]. Hence, extraction or degradation of TC is a critical issue for water control, environmental protection, and methods such as adsorption, biological treatment, photocatalysis, and more have been proposed [5,6,7,8]. Biodegradation is quite promising because organic matters can be converted into inorganic molecules by certain bacteria, but the process is time consuming [9]. In comparison, photocatalysis degradation based on semiconducting materials can harness solar energy for mineralization of organic pollutants and offers advantages such as no secondary pollution, low energy consumption, and practicability. In this respect, semiconducting photocatalysts such as Ag_2_MoO_4_ [10], Ag_3_PO_4_ [11], BiOI [12], Bi_3_O_4_Cl [13], Bi_2_MoO_6_ [14], CdS [15], g-C_3_N_4_ [16], and Ag_2_Se [17] have been proposed for removing organic contaminants [18].

In the interest of ameliorating light-harvesting, transfer and separation of photo-induced carriers, redox reactions, and photocatalytic ability, a direct Z-scheme photocatalysis system has been put forward [19,20,21]. C.Y. Zhou et al. prepared the CCN/Bi_12_O_17_C_l2_ heterojunction for a Z-scheme system and the photocatalytic efficiency for the degradation of tetracycline was enhanced when compared to Bi_12_O_17_C_l2_ [22]. B.S. Li et al. prepared the AgI/Bi_24_O_31_Cl_10_ Z-scheme photocatalyst, which performed better than conventional photocatalysts and 85.36% of TC was degraded under visible light radiation for 60 min [23].

Silver selenide (Ag_2_Se) with a bandgap of 2.6 eV has come into notice due to the good photodegradation properties such as the large redox potential [24], but suffers from drawbacks such as low absorption of visible light, easy photoexcited e^−^/h^+^ pairs recombination, and lack of circulation stability. A viable approach to enhance the photocatalysis ability of Ag_2_Se is to construct a heterojunction with the suitable semiconductors. Cerium dioxide (CeO_2_) has a narrow bandgap, high stability, and high photocatalysis ability boding well for degradation of pollutants. Under reduction conditions, Ce^4+^ ions are reduced to Ce^3+^ to facilitate formation of surface oxygen vacancies [25,26]. Recently, 0-dimensional semiconductor quantum dots (QDs) have been incorporated into photocatalysts, photodetectors, and photo-electric devices [27,28]. In fact, CeO_2_ quantum dots have excellent photocatalysis ability on account of the small size (<10 nm) and quantum confinement effects enable better contact with other atoms in the heterostructure and formation of a better interface between the two components [29]. Moreover, the shorter effective charge transfer length reduces the probability of charge carrier recombination, which renders CeO_2_ QDs suitable for photocatalytic applications.

In this work, a two-step synthesis process encompassing hydrothermal treatment and wet impregnation is designed to produce the CeO_2_ QDs/Ag_2_S Z-scheme nano-heterojunction. Compared with CeO_2_ QDs and Ag_2_Se, CeO_2_ QDs/Ag_2_Se exhibits excellent photocatalytic activity and photostability in TC degradation because the photoinduced e^−^/h^+^ pairs are separated effectively in addition to the high redox ability. The catalytic activity of CeO_2_ QDs/Ag_2_Se for TC can be further enhanced by applying an electric field and the underlying mechanism is investigated.

## 2. Materials and Methods

### 2.1. Preparation of Ag_2_Se Nanoflowers

The solution was formed by adding 1.72 g of Ag_2_Se powder and 1.7 g of ethylenediamine tetra-acetic acid to 30 mL of distilled water and stirred with a glass rod until complete dissolution. Furthermore, 0.4 g of Se powder and 0.13 mL of sodium borohydride were added into the solution, magnetically stirred for 1 h, migrated to a 50 mL autoclave, sealed, and warmed to 160° for 12 h. After the reaction, the precipitation was taken out, centrifuged, rinsed repeatedly, alternated with anhydrous ethanol and deionized water to get rid of impurities and unreacted chemicals, and dried in a vacuum oven at 60° for 6 h.

### 2.2. Formation of CeO_2_ QDs/Ag_2_Se Z-Scheme Nanoheterojunction

Different amounts of CeO_2_ QDs were incorporated into the CeO_2_ QDs/Ag_2_Se samples by changing the molar ratio of Ce/Ag. In addition, 40 mg of Ag_2_Se and 8.87 mg of cerium nitrate hexahydrate were mixed in 40 mL of deionized water and stirred for 60 min. Furthermore, 4 mL of hydrazine hydrate were then added dropwise and stirred for 2 h under nitrogen. After the reaction, the precipitation was washed and centrifuged many times with distilled water and anhydrous ethanol and then dried at 70° to produce the 10% CeO_2_ QDs/Ag_2_Se composites. The CeO_2_ QDs/Ag_2_Se samples with different amounts of CeO_2_ QDs were designated as 5% CeO_2_ QDs/Ag_2_Se, 10% CeO_2_ QDs/Ag_2_Se, 15% CeO_2_ QDs/Ag_2_Se, and 20% CeO_2_ QDs/Ag_2_Se and, for comparison, Ag_2_Se and CeO_2_ QDs were prepared using the same protocol.

### 2.3. Characterization

X-ray power diffraction (XRD, Rigaku D/max-2000, Tokyo, Japan) was employed to characterize the structure of the specimens using Cu K_α_ (*λ* = 1.5406 Å) radiation at 40 KV and 200 mA in a 2*θ* range of 20°–70°. Scanning electron microscopy (SEM, Hitachi S-4800, Tokyo, Japan) was performed to examine the morphology and transmission electron microscopy (TEM) and high-resolution transmission electron microscopy (HR-TEM) were investigated (JEOL JEM-2100F, Tokyo, Japan). The surface chemical composition was investigated using X-ray photoelectron spectra (XPS) (PHI 5000C ESCA System, Tokyo, Japan). Raman scattering was conducted (Jobin Yvon XploRA, Paris, France). UV-vis spectrophotometry (UV-Vis; PerkinElmer Lambda 35, Waltham, MA, USA) was used to test the optical absorption under room temperature and the photoluminescence (PL) characteristics were conducted on fluorescence spectrophotometry (FLS-920, Livingston, Scotland, UK). The Brunauer-Emmett-Teller (BET) specific surface areas were measured (Tristar 3000 nitrogen adsorption apparatus, Norcross, GA, USA).

### 2.4. Photocatalytic Activity Evaluation

Tetracycline (TC) was degraded by CeO_2_ QDs, Ag_2_Se, and CeO_2_ QDs/Ag_2_Se with an 8 W halogen lamp (KLD-08L/P/N, Gyeonggi-do, Korea) as illuminant. A total of 50 mg of the photocatalyst were scattered in 50 mL of a solution with a TC concentration of 0.02 g/L. The beaker was placed in the box in which the lamp was placed 100 mm above the solution. The luminescent efficiency of the lamp was 80 lm/W and the wavelength range was between 400 and 790 nm. Prior to the photocatalytic experiment, the solution was magnetically stirred in the dark for 60 minutes to establish an adsorption-desorption equilibrium. Additionally, 10 ml of the sample were extracted from the solution and spun at 10,000 rpm in the rotator to eliminate the solid materials. Afterward, the lamp was turned on and photocatalytic degradation proceeded under magnetic stirring. The specimens were taken from the beaker at regular intervals, centrifuged for 10 min to remove the powder, and carried out on UV-vis spectrophotometry at 357 nm.

The intermediate products during the degradation of TC were identified using LC-MS (LC-Agilent Technologies 1290 Infinity, Palo Alto, CA, USA; MS-AB SCIEX QTR AP4500, Waltham, MA, USA), ZORBAX Eclipse Plus C18 Column (150 mm × 2.1 mm, 3.5 μm) (Agilent, Santa Clara, CA, USA). During the process, formic acid water: acetonitrile: methanol (45:35:20) was used as a mobile phase. The flow rate was 0.2 mL/min. The mass spectra with a scan range m/z 50–500 were recorded.

### 2.5. Electrocatalytic and Photoelectron Catalytic Activity Assessment

The photocurrents and electrochemical impedance spectra (EIS) were carried out on an electrochemical workstation (CHI 660E, Chenhua Instruments Co., Shanghai, China) based on the standard three-electrode system. The platinum plate was the counter electrode (CE) and a standard calomel electrode was the reference electrode (RE). The electrolyte was 0.1 M sodium sulfate. To prepare the working electrode (WE), 10 mg of the photocatalyst were scattered in 0.5 mL of absolute alcohol with 20 μL of 5 wt% electrolyte and sonicated for 1 h to a uniform suspension. Afterward, 60 μL of the slurry were put on the surface of 1 cm x 1 cm indium tin oxide glass and annealed in air for 2 h at 200 °C.

To monitor the photo-eletrocatalytic activity, the working electrode was immersed in 50 mL of the aqueous solution with a TC concentration of 0.02 g/L. Prior to irradiation, the solution was remained in the dark for 60 min to create adsorption-desorption equilibria. The lamp was turned on and a potential of 0.5 V was applied during the photo-electrocatalytic process. For a comparison, the experiment was also conducted without visible light.

## 3. Results and Discussion

### 3.1. Phase Analysis

**Figure 1:**
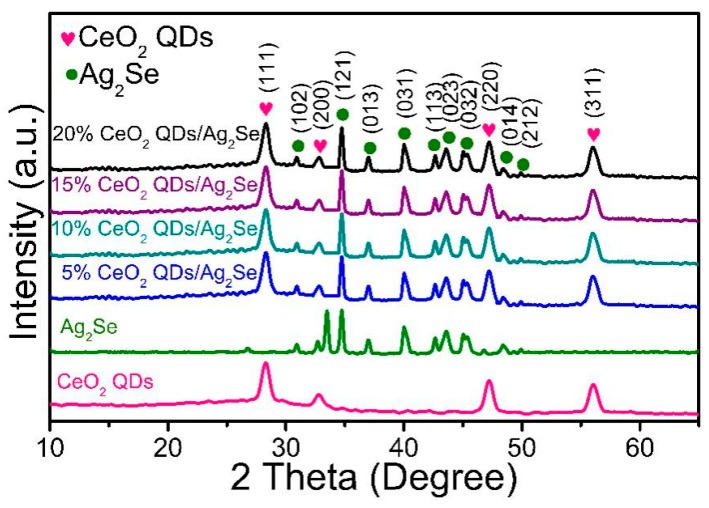
XRD analysis of CeO_2_ QDs, Ag_2_Se, and CeO_2_ QDs/Ag_2_Se with different concentrations of CeO_2_ QDs.

The XRD patterns of CeO_2_ QDs, Ag_2_Se, and CeO_2_ QDs/Ag_2_Se with different amounts of CeO_2_ QDs are shown in Figure 1. There are four strong 2 theta peaks at 28.6°, 33.1°, 47.5°, and 56.4° corresponding to the (111), (200), (220), and (311) planes of the cubic phase CeO_2_ (JCPDS file No. 43-1002). The peaks from Ag_2_Se confirm the orthorhombic crystal planes (JCPDS file No. 24-1041). In addition, the sharp peaks at 30.9°, 34.7°, 36.9°, 39.9°, 42.6°, 43.4°, 45.1°, 48.6°, and 49.9° are assigned to the (102), (121), (013), (031), (113), (023), (032), (014), and (212) planes, respectively. The large intensity and sharpness suggest high crystallinity. The CeO_2_ QDs/Ag_2_Se samples have complex Ag_2_Se and CeO_2_ phases without other peaks, which indicates high purity.

### 3.2. XPS

**Figure 2:**
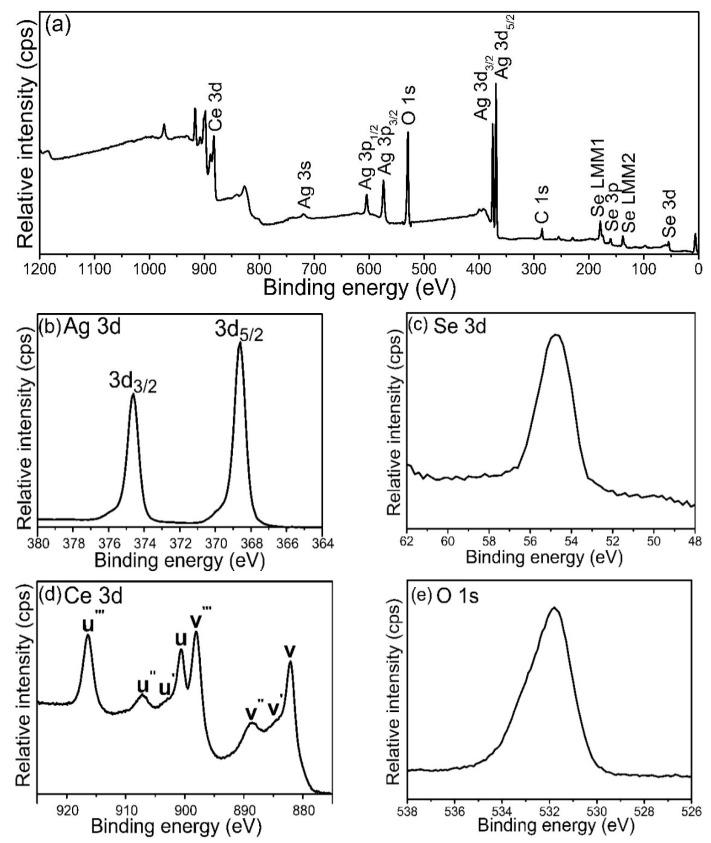
XPS spectra of 10% CeO_2_ QDs/Ag_2_Se: (**a**) Survey scan, (**b**) Ag 3d, (**c**) Se 3d, (**d**) Ce 3d, and (**e**) O 1s.

The element valence states determined by XPS are presented in Figure 2. The XPS spectrum of 10% CeO_2_ QDs/Ag_2_Se shows the Ag 3d, Se 3d, Ce 3d, O 1s, and C 1s peaks in Figure 2a. The C 1s peak originates from impurities and other impurities are not detected. Figure 2b displays two peaks at 375.5 eV and 368.5 eV ascribing to Ag 3d_3/2_ and Ag 3d_5/2_ and the strong peak at 54.8 eV in Figure 2c represents Se 3d in accordance with the archival data of Ag_2_Se [30]. Figure 2d depicts the Ce 3d spectrum, which reveals four pairs of spin orbital dipoles. The peaks located at u′′ (908.4 eV) and v′′ (888.9 eV) represent Ce^3+^ and the other three bimodal peaks, u (900.8 eV) and v (882.4 eV), u′ (903.6 eV), and v′ (884.8 eV), as well as u′′′ (917.1 eV) and v′′′ (898.3 eV), are characteristics of Ce^4+^, which indicates coexistence of Ce^3+^ and Ce^4+^ in the CeO_2_ QDs/Ag_2_Se Z-scheme heterojunction [31]. Figure 2e shows the O 1s peak at 531.8 eV arising from Ce–O in CeO_2_.

### 3.3. Morphological Examination and Reaction Mechanism

**Figure 3:**
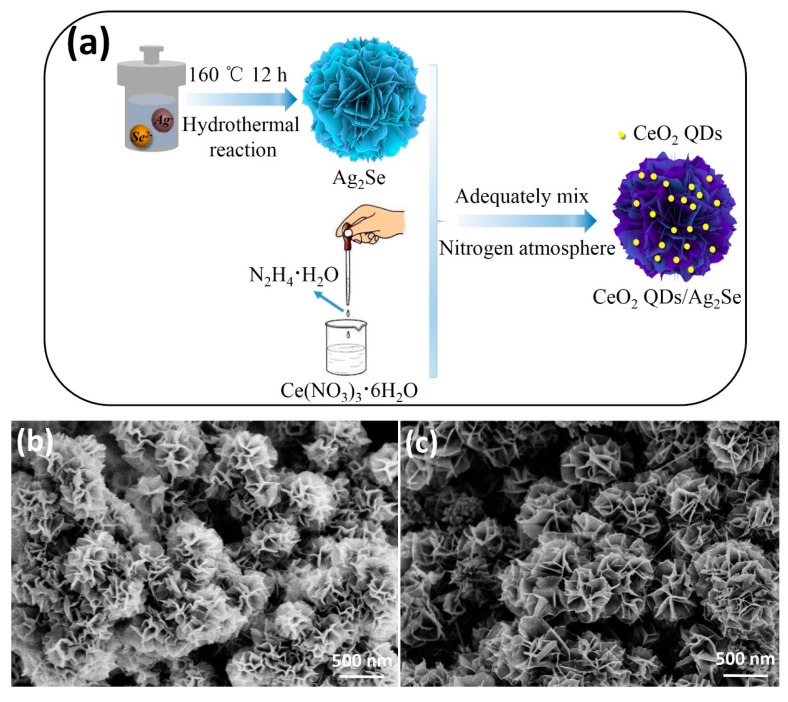
(**a**) Schematic diagram of CeO_2_ QDs/Ag_2_Se Z-scheme nano-heterojunction synthesis. SEM images of (**b**) Ag_2_Se nanoflowers and (**c**) 10% CeO_2_ QDs/Ag_2_Se.

The synthesis process of the CeO_2_ QDs/Ag_2_Se Z-scheme nano-heterojunction and corresponding morphologies are shown in Figure 3a–c, respectively. The Ag_2_Se nanoflowers are prepared hydrothermally at 160 °C for 12 h and Figure 3b reveals an average diameter of about 500 nm. The synthesized Ag_2_Se is then mixed with cerium nitrate hexahydrate and hydrazine hydrate under nitrogen to form the 10% CeO_2_ QDs/Ag_2_Se sample (Figure 3c).

**Figure 4:**
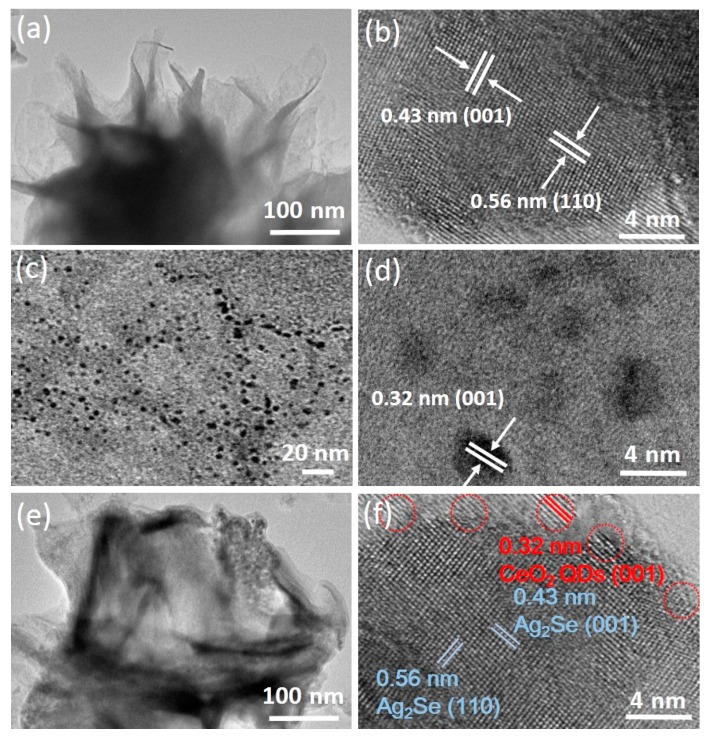
TEM and HR-TEM images: (**a**,**b**) Ag_2_Se nanoflowers, (**c**,**d**) CeO_2_ QDs, and (**e**,**f**) 10% CeO_2_ QDs/Ag_2_Se Z-scheme nano-heterojunction.

**Figure 5:**
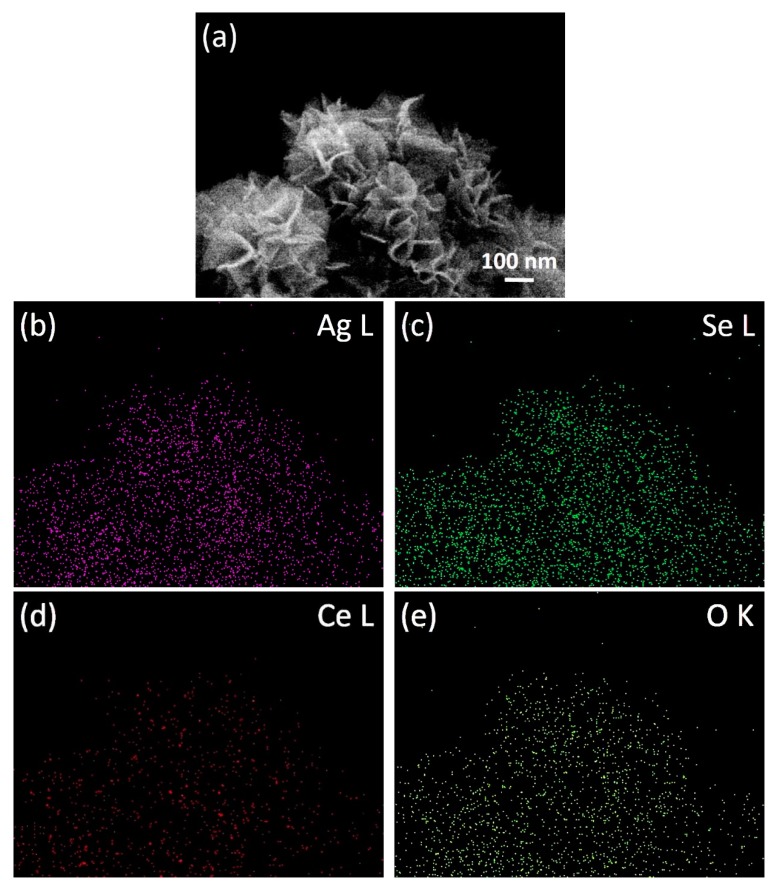
(**a**) SEM image and (**b**−**e**) elemental maps of 10% CeO_2_ QDs/Ag_2_Se.

Figure 4a,c,e display the TEM images of the Ag_2_Se nanoflowers, CeO_2_ QDs, and 10% CeO_2_ QDs/Ag_2_Se, respectively. Figure 4c displays that the average diameter of the sphere is approximately 3.5 nm and, according to Figure 4d, the interplanar distance of the CeO_2_ QDs is 0.32 nm coinciding with the (001) plane of CeO_2_ confirming successful synthesis of CeO_2_ QDs. The morphologies of pure Ag_2_Se nanoflowers and 10% CeO_2_ QDs/Ag_2_Se are not significantly different, as shown in Figure 4a and 4e and Figure 4e shows that the CeO_2_ QDs attach closely to the Ag_2_Se nanoflowers. Figure 4b reveals an interplanar distance of 0.43 nm corresponding to the (001) plane of Ag_2_Se and the interplanar distance of 0.56 nm is associated with the (110) plane of Ag_2_Se. The (001) and (110) planes of Ag_2_Se and (001) plane of CeO_2_ can be observed from Figure 4f, which further corroborates successful fabrication of 10% CeO_2_ QDs/Ag_2_Se. The dark-field TEM (HAADF)-EDS maps show that Ag, Se, Ce, and O are distributed uniformly and Figure 5 shows that CeO_2_ QDs are distributed evenly over the surface of the Ag_2_Se nanoflowers.

### 3.4. Raman Scattering

**Figure 6:**
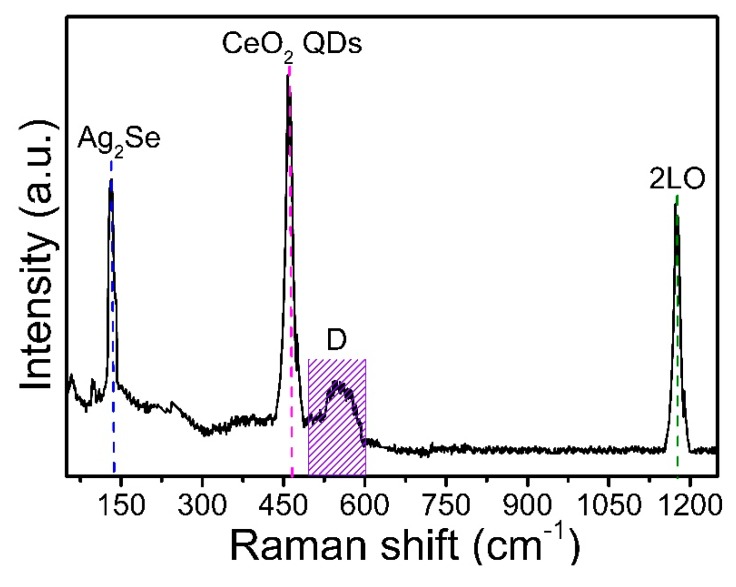
Room-temperature Raman scattering spectrum of 10% CeO_2_ QDs/Ag_2_Se.

Figure 6 represents the room-temperature Raman scattering spectrum of 10% CeO_2_ QDs/Ag_2_Se and the peak at 134 cm^−1^ originates from Ag_2_Se [32]. The other peak at 460 cm^−1^ ascribes to the F_2g_ vibration mode of the fluorite structure of CeO_2_ and the D peaks between 500 and 600 cm^−1^ indicate oxygen vacancies (O_v-b_) in the cerium-fluorite structure [33]. The peak at 1180 cm^−1^ represents the second-order longitudinal optical (2LO) mode. The results show that there are abundant oxygen vacancies over the surface of CeO_2_ QDs to promote the redox reactions and enhance the photocatalytic activity.

### 3.5. Optical Properties

**Figure 7:**
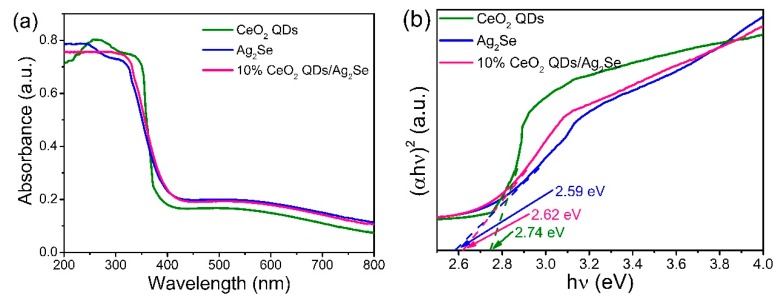
(**a**) UV-visible absorption spectra of CeO_2_ QDs_,_ Ag_2_Se, and 10% CeO_2_ QDs/Ag_2_Se and (**b**) plots of (*αhν*)^2^ versus bandgap (*hν*) of CeO_2_ QDs_,_ Ag_2_Se, and 10% CeO_2_ QDs/Ag_2_Se.

In order to determine the bandgap of CeO_2_ QDs, Ag_2_Se, and 10% CeO_2_ QDs/Ag_2_Se, UV-vis spectrophotometry is performed and the results are presented in Figure 7a,b. The absorption peaks of CeO_2_ QDs at 270 nm represents the transformation from the O 2p state to the Ce 5d state and the steep shape indicates strong absorption caused by the bandgap transition in lieu of impurities [34,35]. Oxygen vacancies in nanocomposites enhance adsorption by acting as electron acceptors to improve charge transport, shift the Fermi level toward the conduction band, and promote charge separation. The samples show high absorption of visible light resulting in the generation of more photo-induced e^−^/h^+^, and improved photocatalytic activity. The bandgaps are calculated according to the Kubelka-Munk equation and the UV-visible absorption spectra of the other samples are displayed in Figure S1 [36]. According to Figure 7b, the bandgaps of CeO_2_ QDs and Ag_2_Se are calculated to be 2.74 and 2.59 eV and those of the other samples are summarized in Table S1. The conduction band $E_{CB}$) and valence band ($E_{VB}$) of the CeO_2_ QDs and Ag_2_Se are calculated according to the following formulas: $E_{VB}=X-E_{C}+0.5E_{g}$ and $E_{CB}=E_{VB}-E_{g}$, where *X* is the electronegativity of the semiconductor (4.875 eV for Ag_2_Se, 5.561 eV for CeO_2_), $E_{C}$ is the energy of free electrons on the atomic scale of hydrogen (approximately 4.5 eV), and $E_{g}$ is the bandgap [37]. $E_{VB}$ and $E_{CB}$ of Ag_2_Se are calculated to be +1.67 eV and −0.92 eV. The valence and conduction bands of the CeO_2_ QDs are +2.43 eV and −0.31 eV, respectively.

**Figure 8:**
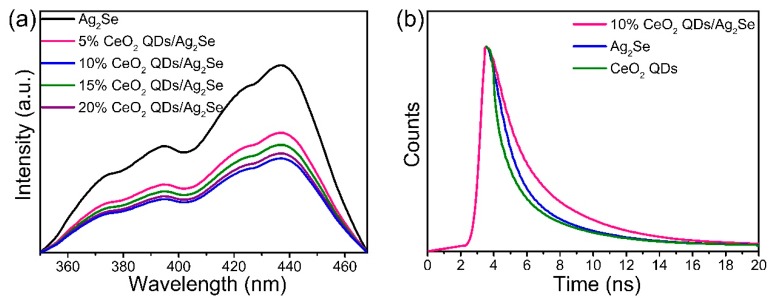
(**a**) Photoluminescence spectra and (**b**) transient PL plots of the different samples.

The recombination rate of photogenerated carriers in CeO_2_ QDs/Ag_2_Se is determined by photoluminescence (PL) excited by the 328 nm laser. Figure 8a shows that the PL intensity from CeO_2_ QDs/Ag_2_Se is significantly less than that from Ag_2_Se, which suggests that the recombination rate of CeO_2_ QDs/Ag_2_Se is smaller and, hence, more photo-induced hole-electron pairs can take part in the redox reactions. Figure 8b displays the transient PL plots of CeO_2_ QDs, Ag_2_Se, and 10% CeO_2_ QDs/Ag_2_Se. The average charge carrier lifetimes of CeO_2_ QDs, Ag_2_Se, and 10% CeO_2_ QDs/Ag_2_Se derived by fitting the curves of three exponential terms are 2.08, 2.13, and 4.08 ns, respectively. The longer life span observed from 10% CeO_2_ QDs/Ag_2_Se stems from rapid separation and transfer of photo-induced carriers.

### 3.6. Photocatalytic Characteristics Under Visible Light Illumination

**Figure 9:**
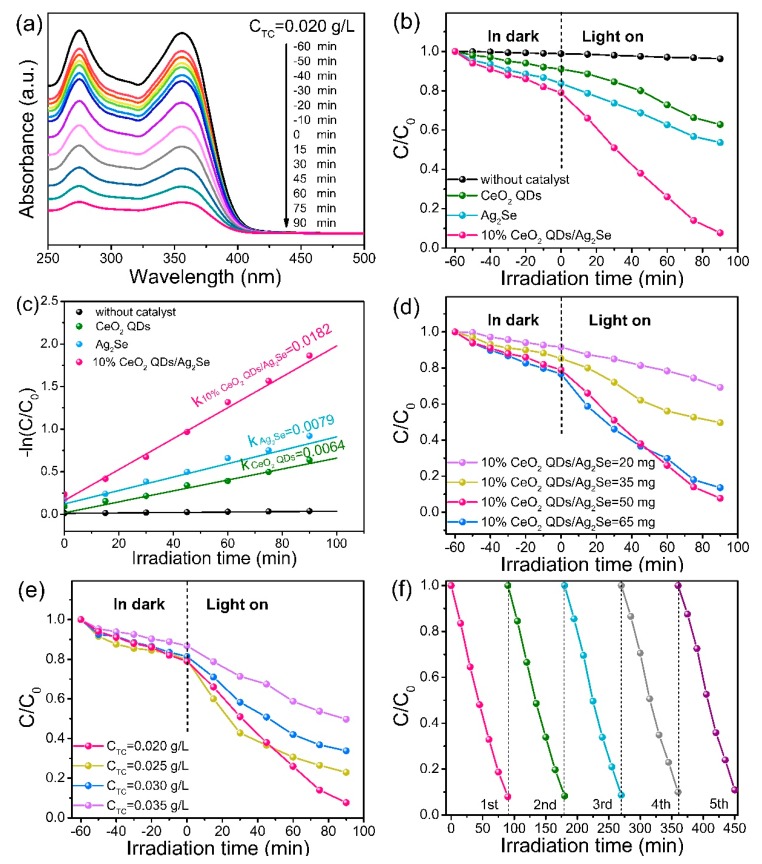
(**a**) Ultraviolet-visible diffuse reflectance spectra for TC photocatalytic degradation by 50 mg of 10% CeO_2_ QDs/Ag_2_Se, (**b**) photodegradation curves of TC, (**c**) Kinetics plots, (**d**) effect of photocatalyst concentrations on TC degradation by 10% CeO_2_ QDs/Ag_2_Se, (**e**) effect of initial concentration of TC on the photocatalysis, and (**f**) cycling tests on photodegradation of TC by 10% CeO_2_ QDs/Ag_2_Se under visible light.

Figure 9a displays the UV-vis absorbance changes of TC in the face of 50 mg of 10% CeO_2_ QDs/Ag_2_Se (initial concentration of TC = 0.02 g/L). The concentration of TC decreases with irradiation time, which confirms the degradation capability of CeO_2_ QDs/Ag_2_Se. The photocatalytic degradation ability of other specimens and kinetics plots are displayed in Figure S2. Among the different samples, 10% CeO_2_ QDs/Ag_2_Se exhibits the best photocatalytic degradation ability toward TC and the corresponding pseudo-first-order rate constant *k* is shown in Table S2. Figure 9b shows the photocatalytic degradation curves of CeO_2_ QDs, Ag_2_Se, and 10% CeO_2_ QDs/Ag_2_Se with and without visible light exposure. Self-degradation of TC can be neglected because it is very stable in the aqueous solution. The results show that 92.3% of TC is degraded by 10% CeO_2_ QDs/Ag_2_Se after 90 min. The first reason is that the CeO_2_ QDs/Ag_2_Se Z-scheme nano-heterojunction has a large specific surface area, which provides abundant reaction sites (The surface area of 10% CeO_2_ QDs/Ag_2_Se is characterized by BET, as shown in Figure S3). The second reason is that the Ce^4+^/Ce^3+^ redox centers give rise to the excellent photo-redox capability. The photocatalytic degradation reaction adheres to the first-order kinetics in general. In the pseudo-first-order reaction, the concentration can be fitted via the equation: −ln(C/C_0_) = *k*t, where *C_0_* is the starting concentration of TC, *C* is the real time concentration of TC during light irradiation, and *k* is the rate constant. Figure 9c reveals that −ln(C/C_0_) is nearly linear with irradiation time, which confirms the first-order kinetics. The change in *k* reflects the variation in the photocatalytic performance. A total of 10% CeO_2_ QDs/Ag_2_Se shows a *k* value of 0.0182 min^−1^ that is about 2.3 times larger than that of Ag_2_Se and 2.84 times bigger than that of the CeO_2_ QDs. The good catalytic degradation characteristics of the CeO_2_ QDs/Ag_2_Se Z-scheme nano-heterojunction are, thus, confirmed.

Figure 9d shows the relationship between TC degradation and photocatalyst concentration. The removal rates of TC by 20 mg of 10% CeO_2_ QDs/Ag_2_Se, 35 mg of 10% CeO_2_ QDs/Ag_2_Se, 50 mg of 10% CeO_2_ QDs/Ag_2_Se, and 65 mg of 10% CeO_2_ QDs/Ag_2_Se are 30.8%, 50.3%, 92.3%, and 86.4%, respectively. The degradation rate of 50 mg of 10% CeO_2_ QDs/Ag_2_Se is the best and a small concentration of the photocatalyst cannot degrade TC completely due to the reduced photocatalytic efficiency. Figure 9e shows the degradation effectiveness of 10% CeO_2_ QDs/Ag_2_Se for different initial TC concentrations. The degradation rates decrease with increasing initial TC concentrations. It is merely 50.2% when the initial TC concentration is 0.035 g/L because degradation of TC depends on the concentration of TC on the photocatalyst surface. A high TC concentration blocks visible light penetration and absorption, which results in degraded photocatalytic performance.

The stability of the photocatalyst is an important parameter and cycling experiments are carried out on 10% CeO_2_ QDs/Ag_2_Se. After the test, the catalyst is collected via centrifugation and rinsed with deionized water and anhydrous alcohol to get rid of residual TC. A new TC solution is added before the next cycle begins and the experimental conditions remain the same for subsequent cycles. As shown in Figure 9f, the catalytic activity of 10% CeO_2_ QDs/Ag_2_Se does not decrease significantly throughout the five cycles and, in fact, the degradation rate after five cycles is still 89.1% compared to 92.3% for the first cycle. Figure S3 reveals no apparent change in 10% CeO_2_ QDs/Ag_2_Se before and after cycling, which suggests good durability and recyclability.

### 3.7. Photoelectrocatalytic Properties of CeO_2_ QDs/Ag_2_Se

**Figure 10:**
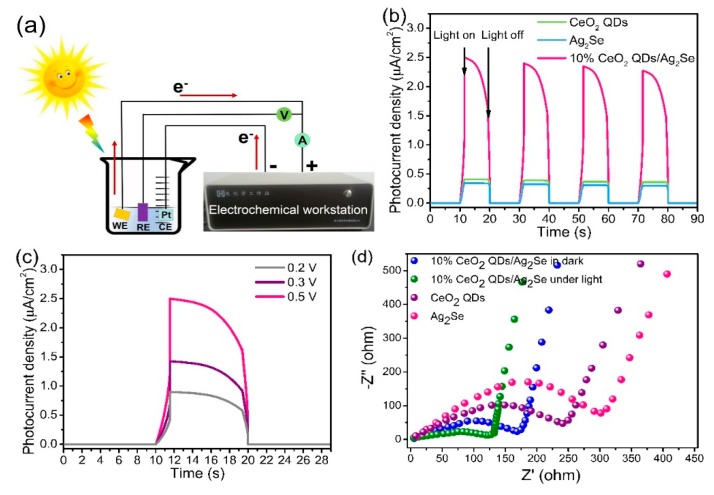
(**a**) Schematic diagram of the test setup, (**b**) transient photocurrent plots of CeO_2_ QDs, Ag_2_Se, and 10% CeO_2_ QDs/Ag_2_Se at 0.5 V *vs.* CE, (**c**) photocurrent plots of 10% CeO_2_ QDs/Ag_2_Se at different biases, (**d**) EIS spectra of CeO_2_ QDs_,_ Ag_2_Se, and 10% CeO_2_ QDs/Ag_2_Se.

CeO_2_ QDs, Ag_2_Se, and 10% CeO_2_ QDs/Ag_2_Se are employed as the working electrodes to evaluate the photo-electrochemical behavior (Figure 10a), and I-T studies are conducted in the 10 s on-off irradiation period at 0.5 V, as shown in Figure 10b. The transient photo-current plots of the samples with different contents of CeO_2_ QDs at 0.5 V (vs. CE) are revealed in Figure S4 and the photo-current of 10% CeO_2_ QDs/Ag_2_Se is 6.13 times that of CeO_2_ QDs and 7.35 times that of Ag_2_Se. When the lamp is turned on, the photocurrent density leaps instantaneously and then stabilizes. When the light is off, the dark current drops rapidly before stabilizing. The photo-current trend repeats during the four on-off cycles, which indicates that CeO_2_ QDs/Ag_2_Se has good stability. The photocurrent of the CeO_2_ QDs/Ag_2_Se Z-scheme nano-heterojunction is bigger than that of CeO_2_ QDs and Ag_2_Se. which suggests CeO_2_ QDs/Ag_2_Se inhibits recombination of photoinduced e^−^/h^+^ pairs. The relationship between the photocurrent and the applied potential is investigated via monitoring the on and off currents at diverse potentials (0.2, 0.3, and 0.5 V vs. RE). As shown in Figure 10c, when the voltage increases from 0.2 to 0.5 V, the photocurrent increases between 0.9 and 2.5 μA cm^−2^. The superior photocurrent indicates that separation of photoelectron-hole pairs by CeO_2_ QDs/Ag_2_Se is more effective at a larger potential. The interfacial conductivity of CeO_2_ QDs, Ag_2_Se, and CeO_2_ QDs/Ag_2_Se is assessed by EIS, as shown in Figure 10d, compared to the Nyquist plots of pure CeO_2_ QDs, and Ag_2_Se. The charge transfer resistance of 10% CeO_2_ QDs/Ag_2_Se is smaller with and without visible light irradiation providing evidence that the surface oxygen vacancies in the CeO_2_ QDs/Ag_2_Se nano-heterojunction facilitate the separation of photon-generated carriers and rapid transport of interfacial electron-hole pairs.

**Figure 11:**
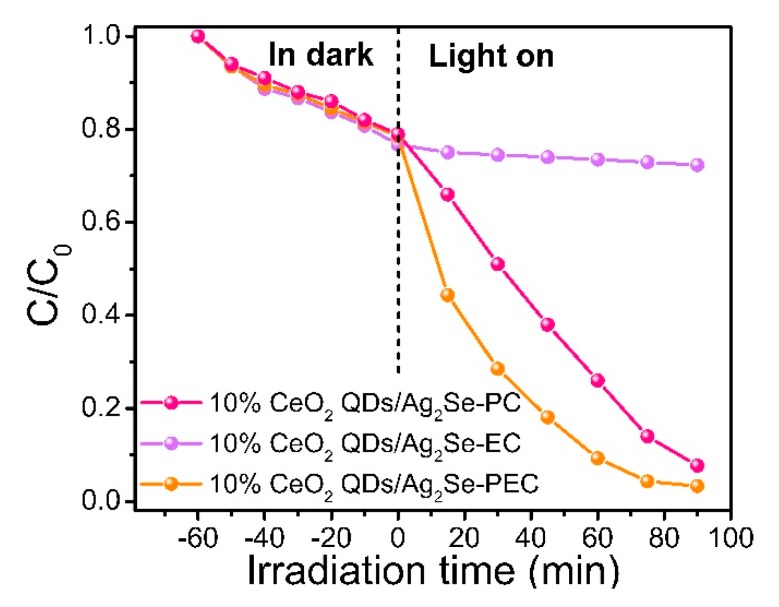
Adsorption and photocatalytic (PC), electrocatalytic (EC), and photo-electrocatalytic (PEC) degradation effects of TC (0.02 g/L, 50 mL) by 10% CeO_2_ QDs/Ag_2_Se Z-scheme nano-heterojunction.

Figure 11 shows the photocatalytic, electrocatalytic, and photo-electrocatalytic effects of 10% CeO_2_ QDs/Ag_2_Se under the same conditions. The adsorption and photo-electrocatalytic (PEC) degradation efficiency of the CeO_2_ QDs/Ag_2_Se specimens with distinct quantities of CeO_2_ QDs is displayed in Figure S5 and 10% CeO_2_ QDs/Ag_2_Se exhibits the best photo-electrocatalytic activity. With 10% CeO_2_ QDs/Ag_2_Se being the photocatalyst, electrocatalyst, or photo-electrocatalyst, the absorption activity reaches 18.5%. In the electrocatalytic process, 27.7% of TC is removed by 10% CeO_2_ QDs/Ag_2_Se after 90 minutes under visible light irradiation. In photocatalysis, 92.3% of TC is removed after 90 min and, by applying a 0.5 V potential, 95.8% of TC is degraded by photo-electrocatalytically after 75 min. The results prove that the degradation efficiency of CeO_2_ QDs/Ag_2_Se is improved by applying an electric field.

**Figure 12:**
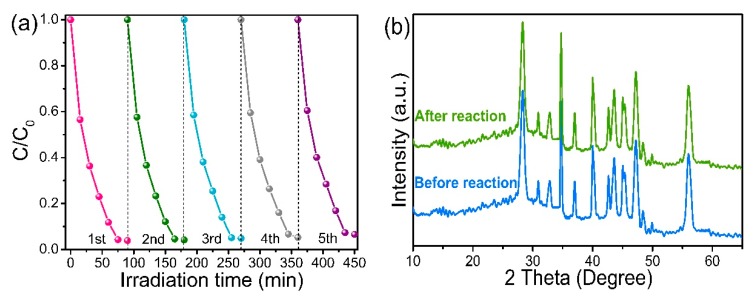

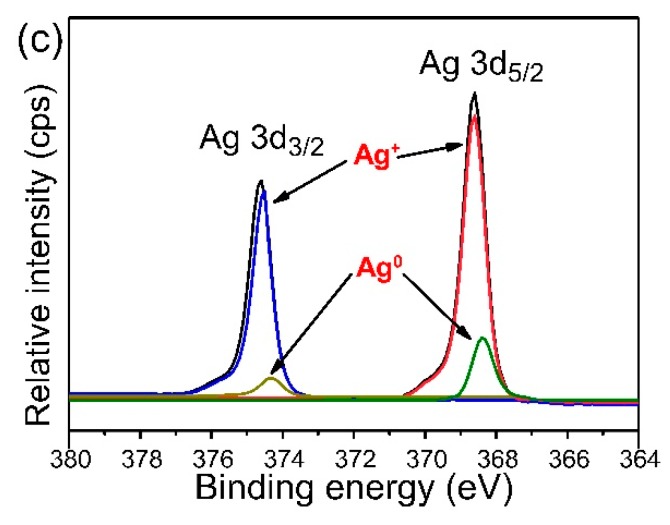
(**a**) Cycling experiments for photo-electrocatalytic degradation on TC by 10% CeO_2_ QDs/Ag_2_Se, (**b**) XRD, and (**c**) Ag 3d XPS spectra of 10% CeO_2_ QDs/Ag_2_Se after five photo-electrocatalytic degradation cycles.

The stability and reusability of CeO_2_ QDs/Ag_2_Se in photo-electrocatalytic degradation are important for practical application. Cycling experiments are carried out on 10% CeO_2_ QDs/Ag_2_Se (Figure 12a). After five cycles, the photo-electrocatalytic activity of 10% CeO_2_ QDs/Ag_2_Se does not decrease significantly and, in the fifth cycle, the photo-electrocatalytic degradation efficiency is still 92.7% (compared to 95.8% in the first cycle). To identify the stability of the crystalline structure after photo-electrocatalytic degradation, the XRD patterns of 10% CeO_2_ QDs/Ag_2_Se before and after five cycles are depicted in Figure 12b. The structure of 10% CeO_2_ QDs/Ag_2_Se does not change significantly, which corroborates good stability and recyclability. To further evaluate the surface component and composition of 10% CeO_2_ QDs/Ag_2_Se, Ag 3d XPS spectra after the photo electrocatalytic reaction are given in Figure 12c. Figure 12c presents the high-resolution Ag 3d spectrum, which is further resolved into four peaks. The peaks located at binding energy 368.07 and 374.07eV are assigned to Ag^+^ in Ag_2_Se [38]. The peaks at 368.31 and 374.33eV are ascribed to Ag 3d_5/2_ and Ag 3d_3/2_, which suggests that part of the silver ions are reduced to Ag [39].

### 3.8. Photocatalytic Mechanisms

**Figure 13:**
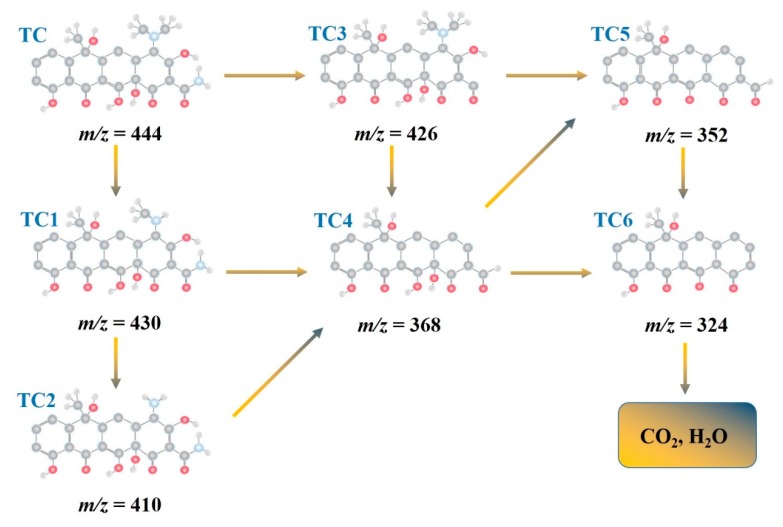
Proposed photocatalytic degradation pathway for TC degradation by CeO_2_ QDs/Ag_2_Se.

The photocatalytic degradation pathway is considered by confirming the degradation intermediates that came into being in the course of photocatalytic degradation and the TC degradation intermediates are determined by LC-MS. MS spectra of TC and possible intermediates at different reaction times were presented in Figure S6. The peak with *m/z* of 444 originates from the TC molecules, whose intensity gradually weakened with the proceeding of the degradation, which indicates the destruction of TC. Peaks of intermediates began to appear at 45-min irradiation periods (Figure S6b). Intensity of some intermediates with *m/z* of 324, 368, 410, and 430 attenuated, which demonstrates that these intermediates were further degraded. Moreover, when the illumination time was extended to 90min, some new intermediates such as *m/z* of 352 and 426 emerged (Figure S6c), which depicted the further degradation process of TC. The pathway of photocatalytic degradation on TC via CeO_2_ QDs/Ag_2_Se is revealed in Figure 13. Irradiation forms active substances such as •OH, •O_2_^−^, and h^+^, which attack the tetracycline molecules and intermediates. Since the N–C bond is weak [40], it is prone to attack and the tetracycline molecules undergo N-demethylation to form the TC1 (*m/z*= 430) and TC2 (*m/z*= 410) intermediates. The original TC molecule undergoes deamination, deamidation, and dihydroxylation to form TC3 (*m/z*= 426), TC4 (*m/z*= 368), and TC5 (*m/z*= 352). TC5 is converted to TC6 (*m/z*= 324) after removing the aldehyde group and, after further degradation, these intermediates are transformed into water, CO_2_, and other organic outcomes.

**Figure 14:**
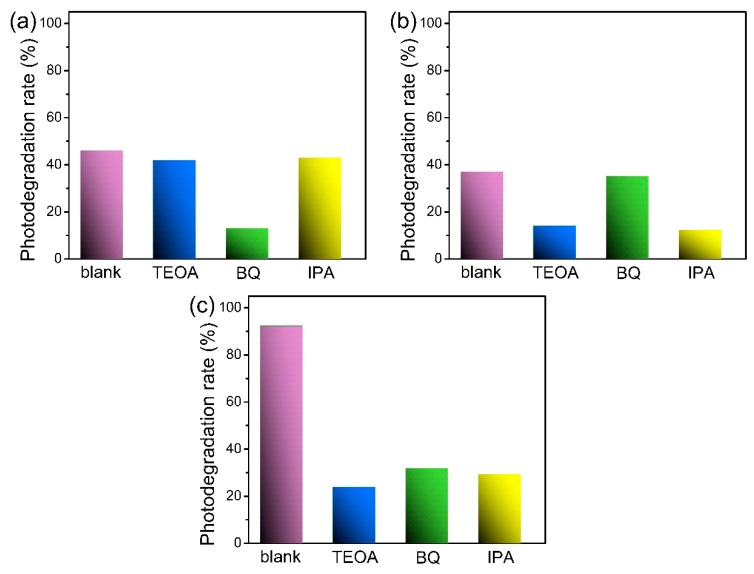
Determination of active species in degradation of TC by (**a**) Ag_2_Se, (**b**) CeO_2_ QDs, and (**c**) 10% CeO_2_ QDs/Ag_2_Se.

To corroborate the formation of the Z-scheme nano-heterojunction, the active species in photocatalytic degradation are studied because the active materials determine the source of electrons and holes. Free radical capture experiments are performed by adding scavengers to the TC solution, as shown in Figure 14. Generally, triethanolamine (TEOA), benzoquinone (BQ), and isopropanol (IPA) are used to capture holes (h^+^), superoxide radical anions (•O_2_^−^), and hydroxyl radicals (•OH), respectively. When Ag_2_Se is the catalyst, the photocatalytic activity changes slightly after the addition of TEOA and IPA. However, when BQ is introduced, the photodegradation rate of TC decreases, which clearly indicates that •O_2_^−^ is the primary activated radical during photodegradation of TC by Ag_2_Se and •OH and h^+^ do not contribute significantly to photocatalytic degradation of TC. When the CeO_2_ QDs serve as the photocatalyst, the photocatalytic activity varies slightly after the addition of BQ. However, when IPA and TEOA are introduced, the photocatalytic degradation rate decreases appreciably, which suggests that •OH and h^+^ are the primary activated radicals on the CeO_2_ QDs. After TEOA, BQ, and IPA are introduced, the photodegradation rate of 10% CeO_2_ QDs/Ag_2_Se decreases precipitously, which implies that h^+^, •O_2_^−^, and •OH play similar roles in the photodegradation of TC.

**Scheme 1:**
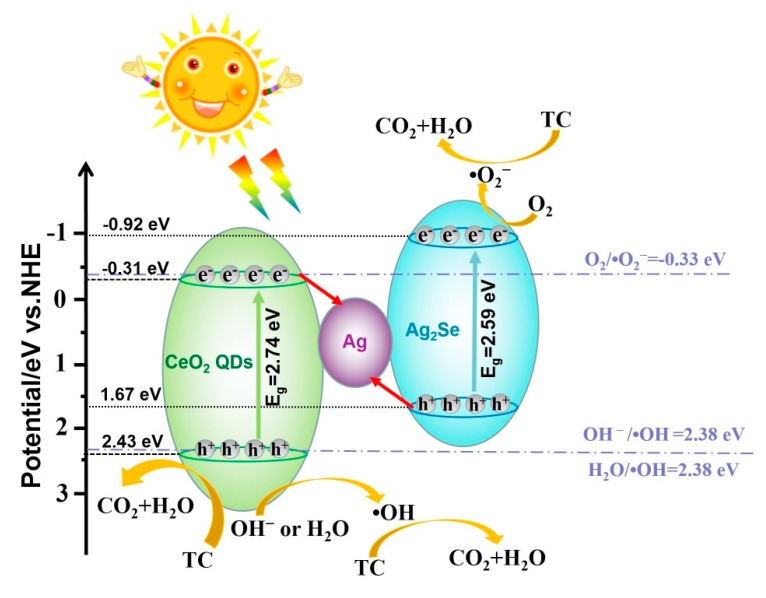
Schematic illustration of electron transfer and active radical synthesis of the CeO_2_ QDs/Ag_2_Se Z-scheme nano-heterojunction under visible light illumination.

According to the energy levels, the probable interface electron transfer behavior is presented in Scheme 1. The potentials of the conduction band and valance band of the CeO_2_ QDs are −0.31 eV and 2.43 eV, respectively, and those of Ag_2_Se are −0.92 eV and 1.67 eV, respectively. •O_2_^−^ is produced by oxygen reduction and the redox potential is −0.33. •OH is formed by OH^−^ or H_2_O at a redox potential of 2.38 V. The redox potential of •O_2_^−^ is −0.33 V, which is more positive than the CB (conduction band) of Ag_2_Se and more negative than the CB of the CeO_2_ QDs. Therefore, •O_2_^−^ is generated by Ag_2_Se but not CeO_2_ QDs. In contrast, the redox potential of •OH of 2.38 V is more positive than the VB (valence band) of Ag_2_Se and more negative than the VB of CeO_2_ QDs, which indicates that •OH is created by the CeO_2_ QDs but not Ag_2_Se. If the CeO_2_ QDs/Ag_2_Se is a type-II nano-heterojunction, the photo-excited electrons will transfer from the conduction band of Ag_2_Se to the conduction band of the CeO_2_ QDs and h^+^ will transfer from the valance band of the CeO_2_ QDs to the valance band of Ag_2_Se. Therefore, e^−^ and h^+^ are shifted to the conduction band of CeO_2_ QDs and valance band of Ag_2_Se, which means that •O_2_^−^ and •OH are not produced in the photocatalytic reaction. However, three activated substances coexist in the photocatalytic degradation process, which suggests e^−^ leaves the conduction band of Ag_2_Se and h^+^ is retained in the valance band of the CeO_2_ QDs. Therefore, the CeO_2_ QDs/Ag_2_Se composite is a direct Z-scheme nano-heterojunction. In the CeO_2_ QDs/Ag_2_Se Z-scheme nano-hetero-structure, the e^−^ accumulates on the conduction band of Ag_2_Se, which reduces a fraction of silver ions to metallic Ag. This forms between Ag_2_Se and CeO_2_ QDs. Since the size of CeO_2_ QDs is small and the contact surface with Ag_2_Se is limited, the amount of metallic Ag formed is small, which can avoid the deposition of extra Ag on the Ag_2_Se surface to hinder the penetration and absorption of visible light and affect the photocatalytic effect. Ag can be used as electronic medium to change the charge carrier transport mode, which is more suitable for the Z-scheme. As shown in Scheme 1, Ag nanoparticles can be used as a charge transfer bridge to form the CeO_2_ QDs/Ag/Ag_2_Se Z-scheme system. Since the conduction band of CeO_2_ QDs is more negative than the Fermi energy of Ag, the photogenerated e^−^ in the conduction band of CeO_2_ QDs move to Ag. At the same time, the h^+^ in the valance band of Ag_2_Se move toward Ag and coalesce with the e^−^. This kind of electronic transportation can effectively enhance the separation of e^−^ and h^+^, and make the e^−^ and h^+^ stay in the conduction band of Ag_2_Se and valance band of CeO_2_ QDs, respectively. It can successfully inhibit the photo corrosion of Ag_2_Se and CeO_2_ QDs, which contributes to the structural stability of CeO_2_ QDs and Ag_2_Se. The redox centers of Ce^4+^/Ce^3+^ formed in CeO_2_ QDs/Ag_2_Se accelerate the following reactions.

Ce^4+^ + e^−^ → Ce^3+^

Ce^3+^ + O_2_ → Ce^4+^ + O_2_^−^

The rapid cycling process not only expedites electron transfer, but also hinders electron-hole pair combination, which consequently improves the photocatalytic ability. It is also noted that transfer and separation of the photo-induced e^−^/h^+^ pairs are more efficacious in the presence of an applied electric field.

## 4. Conclusions

The CeO_2_ QDs/Ag_2_Se Z-scheme nano-heterojunction is prepared by hydrothermal treatment and wet impregnation. Incorporation of CeO_2_ QDs improves the redox ability and promotes charge separation. Under visible light, the photocatalytic degradation on TC by 10% CeO_2_ QDs/Ag_2_Se is 2.5 times that of CeO_2_ QDs and twice that of Ag_2_Se. The CeO_2_ QDs/Ag_2_Se nano-heterojunction shows a clear photocurrent response and high photo-electrocatalytic performance compared to the single photocatalytic or electrocatalytic process. The main reason is that, in the Z-type system including Ag_2_Se, Ag, and CeO_2_ QDs, Ag is used as electronic transfer bridges to efficaciously separate the photogenerated carriers. By transferring the photo-induced holes and electrons to Ag, the photo corrosion of Ag_2_Se and CeO_2_ QDs is successfully inhibited, and the photocatalytic stability of the catalyst is enhanced. Moreover, the surface oxygen vacancies in the CeO_2_ QDs ameliorate separation of photogenerated e^−^/h^+^ pairs. Transfer and separation of photogenerated e^−^/h^+^ pairs are more efficient with an applied electric field. Our results show that the CeO_2_ QDs/Ag_2_Se Z-scheme nano-heterojunction is a convenient, recyclable, efficient, and stable photoelectrode for degradation of organic contaminants.

## Supplementary Materials

The following are available online at https://www.mdpi.com/2079-4991/10/2/253/s1, Figure S1: UV-visible absorption spectra of CeO_2_ QDs_,_ Ag_2_Se, and CeO_2_ QDs/Ag_2_Se (a). Plots of (*αhν*)^2^ versus bandgap (*hν*) of CeO_2_ QDs_,_ Ag_2_Se, and CeO_2_ QDs/Ag_2_Se (b). Table S1: Bandgaps of CeO_2_ QDs, Ag_2_Se, and CeO_2_ QDs/Ag_2_Se. Figure S2: Photocatalytic degradation rates of TC (a). Pseudo-first-order reaction kinetics curves (b). Table S2. *k* values of CeO_2_ QDs, Ag_2_Se, and CeO_2_ QDs/Ag_2_Se. Figure S3: Nitrogen adsorption/desorption isotherms of 10% CeO_2_ QDs/Ag_2_Se. Figure S4: Transient photocurrent evolution of the CeO_2_ QDs/Ag_2_Se composites with different amounts of CeO_2_ QDs in the CeO_2_ QDs/Ag_2_Se (0.5 V *vs.* CE). Figure S5: Adsorption and photo-electrocatalytic (PEC) degradation efficiency of the TC solution (0.02 g/L, 50 mL) in the presence of CeO_2_ QDs/Ag_2_Se composites with different concentrations of CeO_2_ QDs. Figure S6: LC-MS spectra of possible intermediates of TC at different photocatalytic time.
