# Supplementary material for: Ag as Cocatalyst and Electron-Hole Medium in CeO2 QDs/Ag/Ag2Se Z-scheme Heterojunction Enhanced the Photo-Electrocatalytic Properties of the Photoelectrode
Source: Nanomaterials (Basel). 2020 Jan 31;10(2):253. doi: 10.3390/nano10020253 (PMC7075152; doi:10.3390/nano10020253)
Supplement: Supplementary file 1 [file nanomaterials-10-00253-s001.pdf]

Supplementary Information

Ag as Cocatalyst and Electron-Hole Medium in CeO_2 QDs/Ag/Ag_2Se Z-scheme Heterojunction Enhanced the Photo-Electrocatalytic Properties of the Photoelectrode

Lingwei Li [1], Hange Feng [1], Xiaofan Wei [1], Kun Jiang [1], Shaolin Xue [1,*] and Paul K. Chu [2,*]

1 College of Science, Donghua University, Shanghai 201620, China; li.lingwei.pink@163.com (L.L.); 17721486315@163.com (H.F.); 13683727604@163.com (X.W.); jiangkun95@163.com (K.J.)

2 Department of Physics, Department of Materials Science and Engineering, and Department of Biomedical Engineering, City University of Hong Kong, Tat Chee Avenue, Kowloon, Hong Kong, China

* Correspondence: slxue@dhu.edu.cn (S.X.); paul.chu@cityu.edu.hk (P.K.C.); Fax: +86-216-779-2089 (S.X.); Fax: +852-3442-0538 (P.K.C.)

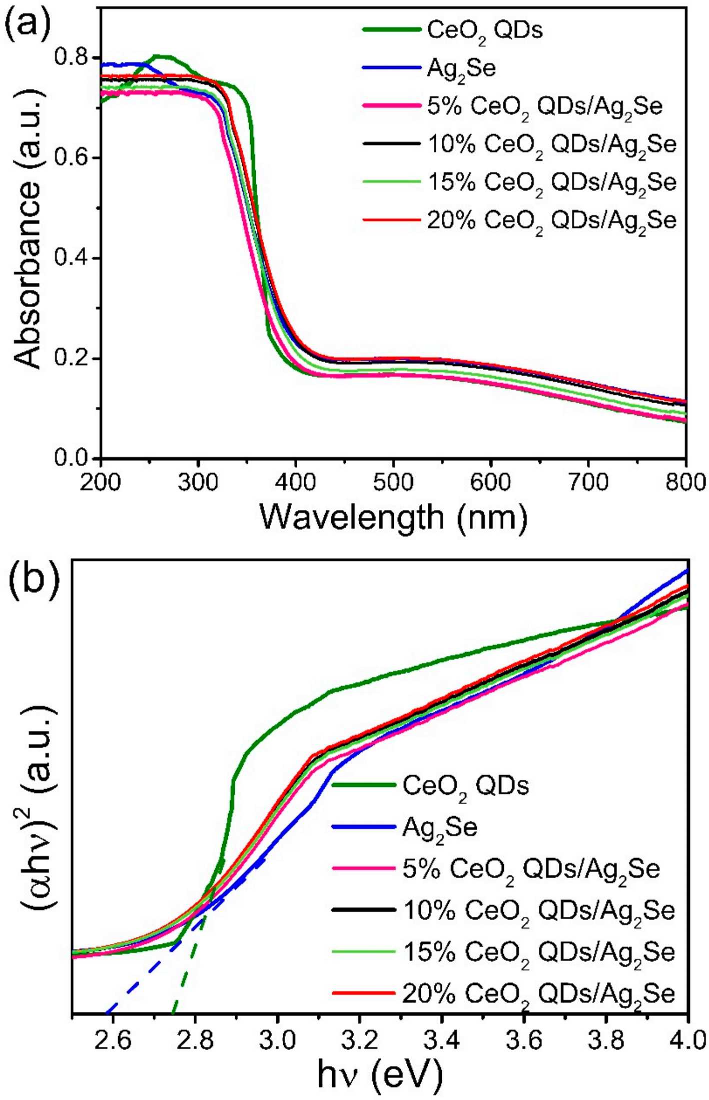

Figure S1. (a) UV-visible absorption spectra of CeO_2 QDs, Ag_2Se, and CeO_2 QDs/Ag_2Se and (b) Plots of $(\alpha h\nu)^2$ versus bandgap ($h\nu$) of CeO_2 QDs, Ag_2Se, and CeO_2 QDs/Ag_2Se.Fig. S1 shows that CeO_2 QDs/Ag_2Se exhibits broad light absorption and as the concentration of CeO_2 QDs goes up, the absorption edge lengthens gradually indicating decreasing bandgaps.

Figure S1 shows that CeO_2 QDs/Ag_2Se exhibits broad light absorption and as the concentration of CeO_2 QDs goes up, the absorption edge lengthens gradually indicating decreasing bandgaps.

Table S1. Bandgaps of CeO_2 QDs, Ag_2Se, and CeO_2 QDs/Ag_2Se.

**Samples**	**Bandgaps (eV)**
CeO_2 QDs	2.74
5% CeO_2 QDs/Ag_2Se	2.66
10% CeO_2 QDs/Ag_2Se	2.62
15% CeO_2 QDs/Ag_2Se	2.64
20% CeO_2 QDs/Ag_2Se	2.61
Ag_2Se	2.59

Figure S2 shows the photocatalytic properties of the CeO_2 QDs/Ag_2Se composites containing different amounts of CeO_2 QDs. Compared to Ag_2Se and CeO_2 QDs, degradation of TC is improved by the CeO_2 QDs/Ag_2Se Z-scheme heterojunctions. The photocatalytic activities follow the order of CeO_2 QDs < Ag_2Se < 5% CeO_2 QDs/Ag2Se < 15% CeO_2 QDs/Ag_2Se < 20% CeO_2 QDs/Ag_2Se < 10% CeO_2 QDs/Ag_2Se and 10% CeO_2 QDs/Ag_2Se shows the largest reaction rate constant (0.0182 min^{-1}).

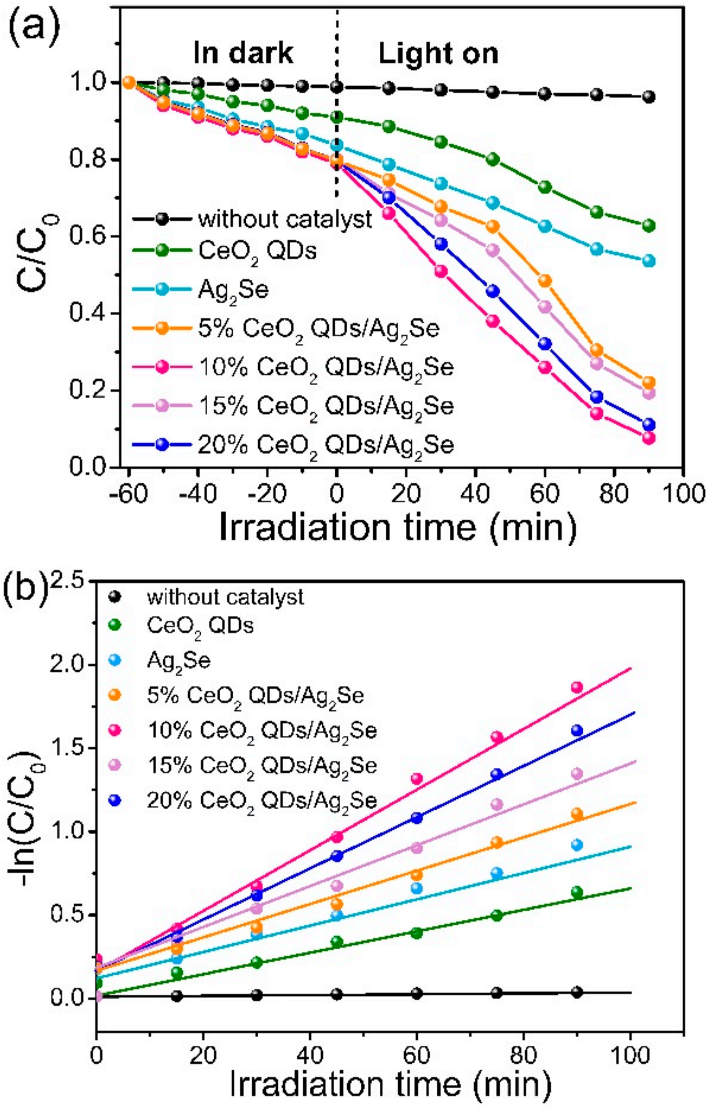

Figure S2. (**a**) Photocatalytic degradation rates of TC and (**b**) Pseudo-first-order reaction kinetics curves.

Table S2. *k* values of CeO_2 QDs, Ag_2Se, and CeO_2 QDs/Ag_2Se.

Samples	*k* values (min^{-1})
CeO_2 QDs	0.0064
5% CeO_2 QDs/Ag_2Se	0.0147
10% CeO_2 QDs/Ag_2Se	0.0182
15% CeO_2 QDs/Ag_2Se	0.0158
20% CeO_2 QDs/Ag_2Se	0.0171
Ag_2Se	0.0079

Figure S3 shows the nitrogen adsorption isotherms of 10% CeO_2 QDs/Ag_2Se. On the basis of the Brunauer-Deming-Deming-Teller (BDDT) classification, the isotherm belongs to the IV type. The characteristic of type IV isotherm is its hysteresis loop, which confirms the existence of mesopores on the surface of the samples. The initial part of the IV isotherm, that is, slightly deflecting the Y axis at the low pressure end, indicates that the material and the nitrogen have a strong force, which is attributed to the monolayer adsorption and the inflection point indicates the saturated adsorption of the single molecular layer. In the middle pressure section (p/p_0 = 0.45–0.60), the adsorption capacity increases slowly. At this point, nitrogen molecules are adsorbed on the inner surface of the mesoporous from monolayer to multilayer. The adsorption capacity of the high pressure section (p/p_0 = 0.7–1) increases abrupt and the starting point of the hysteresis loop indicates that the smallest fine hole begins to coacervation, and the end of the hysteresis loop indicates that the largest hole is filled with the condensed liquid. The specific surface area of 10% CeO_2 QDs/Ag_2Se is about 33.98 m^2/g.

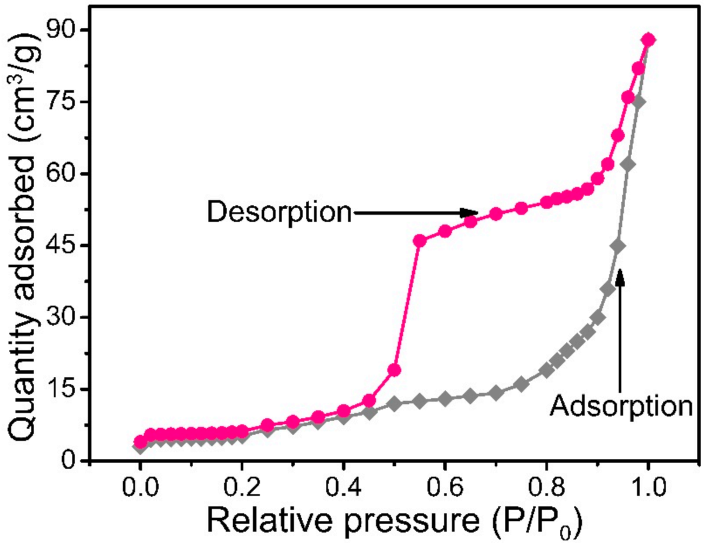

Figure S3. Nitrogen adsorption/desorption isotherms of 10% CeO_2 QDs/Ag_2Se.

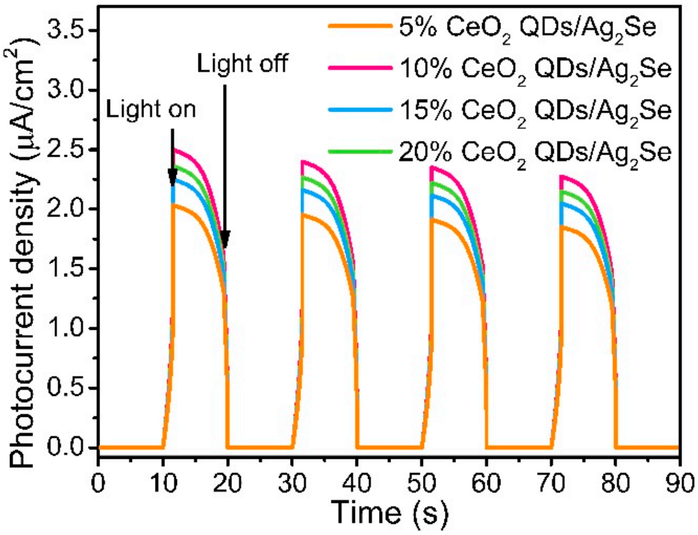

Figure S4. Transient photocurrent evolution of the CeO_2 QDs/Ag_2Se composites with different amounts of CeO_2 QDs in the CeO_2 QDs/Ag_2Se (0.5 V *vs.* CE).

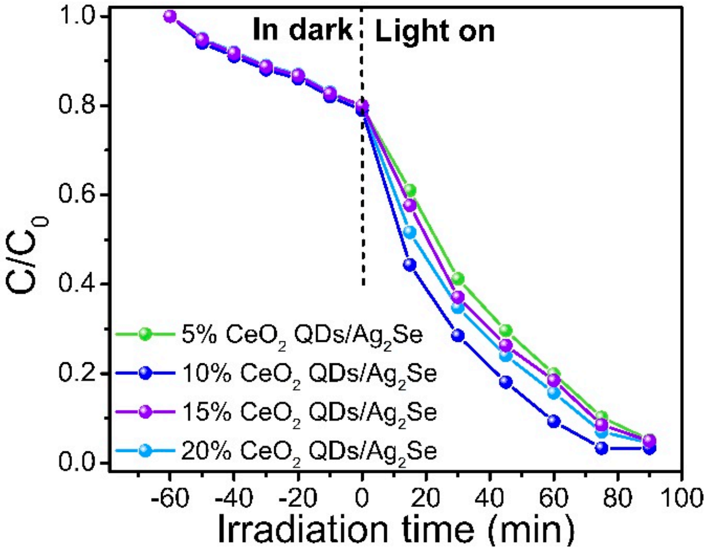

Figure S5. Adsorption and photoelectrocatalytic (PEC) degradation efficiency of the TC solution (0.02 g/L, 50 mL) in the presence of CeO_2 QDs/Ag_2Se composites with different concentrations of CeO_2 QDs.

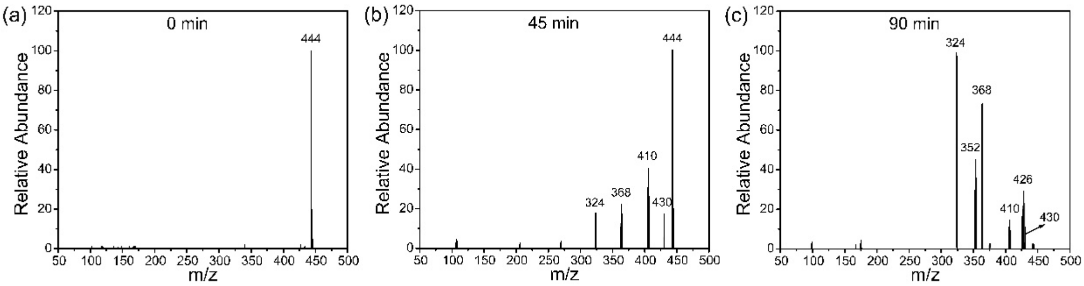

Figure S6. LC-MS spectra of possible intermediates of TC at different photocatalytic time.
